# Antennae hold a key to *Varroa*-sensitive hygiene behaviour in honey bees

**DOI:** 10.1038/srep10454

**Published:** 2015-05-22

**Authors:** Fanny Mondet, Cédric Alaux, Dany Severac, Marine Rohmer, Alison R. Mercer, Yves Le Conte

**Affiliations:** 1 INRA, UR 406 Abeilles et Environnement, 84914 Avignon Cedex 09, France; 2 Department of Zoology, University of Otago, Dunedin 9054, New Zealand; 3 AgroParisTech, 75005 Paris, France; 4 MGX – Montpellier GenomiX, Institut de Génomique Fonctionnelle, 141 rue de la Cardonille, 34094, Montpellier Cedex 05, France

## Abstract

In honey bees, *Varroa* sensitive hygiene (VSH) behaviour, which involves the detection and removal of brood parasitised by the mite *Varroa destructor*, can actively participate in the survival of colonies facing *Varroa* outbreaks. This study investigated the mechanisms of VSH behaviour, by comparing the antennal transcriptomes of bees that do and do not perform VSH behaviour. Results indicate that antennae likely play a key role in the expression of VSH behaviour. Comparisons with the antennal transcriptome of nurse and forager bees suggest that VSH profile is more similar to that of nurse bees than foragers. Enhanced detection of certain odorants in VSH bees may be predicted from transcriptional patterns, as well as a higher metabolism and antennal motor activity. Interestingly, Deformed wing virus/*Varroa destructor* virus infections were detected in the antennae, with higher level in non-VSH bees; a putative negative impact of viral infection on bees’ ability to display VSH behaviour is proposed. These results bring new perspectives to the understanding of VSH behaviour and the evolution of collective defence by focusing attention on the importance of the peripheral nervous system. In addition, such data might be useful for promoting marker-assisted selection of honey bees that can survive *Varroa* infestations.

## Introduction

The honey bee parasite *Varroa destructor* is currently considered to represent the greatest threat to the beekeeping industry worldwide^1^. This parasite infests honey bee colonies and reproduces within capped brood cells containing developing bees. Parasitised individuals are weakened due to feeding on honey bee haemolymph, which triggers – directly or through virus transmission - a large number of physiological responses that lead ultimately to a severe reduction in the lifespan of the bee^2^,^3^,^4^. Development of *Varroa* populations within honey bee colonies, if left untreated, often leads the death of the colony^5^. The parasite has been unambiguously shown to participate in the large-scale periodical colony losses reported worldwide^6^.

Despite the profound impact of *Varroa* parasitism on honey bee populations, honey bees in several parts of the world have been shown to successfully survive the pathogen^7^,^8^,^9^. This strongly suggests that some bees are able to utilise defence mechanisms to fight successfully against mite infestation. In honey bee colonies that survive the mite, *Varroa* sensitive hygiene (VSH) behaviour has been shown to participate actively in limiting outbreaks of *Varroa* parasitisation. This form of hygienic behaviour involves the detection and removal of brood parasitised by *Varroa*^10^ and it contributes to a social immunity repertoire^11^; a new immune repertoire level that evolved with sociality and that includes the use of antimicrobial materials for nest construction^12^, social fever^13^ and nest hygiene and grooming^14^,^15^.

Several honey bee lines have been successfully bred using VSH performance as the main selection criterion, and these colonies show a high level of resistance to *Varroa*^10^,^16^,^17^. Bees from these colonies display effective removal of *Varroa*-infested developing bees from capped brood cells, which in turn seems to limit *Varroa* infestation rate and to reduce the reproduction success of the mite^18^.

Breeding honey bee colonies that are resistant to *Varroa* currently represents the main hope for a long-term solution to fight infestations by *Varroa destructor*, and it provides a promising alternative to acaricide treatments since this selection strategy would favour mechanisms that contribute to a stable host-parasite relationship. While several studies have explored the mechanisms of VSH and general hygienic behaviour^19^,^20^,^21^, the molecular basis and functional pathways involved in VSH behaviour remain partly unknown. Identification of genes that influence VSH behaviour would help reveal the mechanisms that underpin this form of social immunity, and more importantly, provide potential targets for marker-assisted selection of *Varroa* resistant honey bee colonies.

Several studies have shown that olfaction plays a role in hygienic behaviour^22^,^23^. This suggests that VSH bees have the ability to detect the presence of *Varroa* through the cap of parasitised brood cells using olfactory cues. If so, the antennae, which house a diverse array of sensory structures including olfactory sensilla^24^ are likely to play a critical role in the initiation of VSH behaviour. In many insects, physiological and neuroanatomical changes at the level of the peripheral sense organs influence the behavioural state of individuals^25^,^26^,^27^, and it has been suggested that changes in gene expression at the level of the antennae can contribute to shifts in the behavioural states of honey bees^28^. Based on such evidence, it is hypothesised here that functional properties of the antennae play a key role in the ability of adult honey bees to develop VSH behaviour in response to *Varroa* infestation of the brood.

A bioassay is described that enables adult bees performing VSH related tasks (i.e. opening and/or removing *Varroa* parasitised capped brood cells) to be identified within a given honey bee colony, as well as bees that do not perform such tasks. In an attempt to find a genetic signature for VSH behaviour, a transcriptomic approach was used to identify genes that are differentially expressed in the antennae of VSH and non-VSH honey bees. The differential gene expression pattern from these two groups was compared to the pattern obtained from a comparison of antennal transcriptomes from forager and nurse bees. Results provide novel clues about the mechanisms that underlie VSH and candidate genes are identified that have the potential to be used as targets for marker-assisted selection in breeding programs for *Varroa* resistant bees.

## Results

### Age and behavioural characteristics of honey bees performing VSH tasks

**Figure 1:**
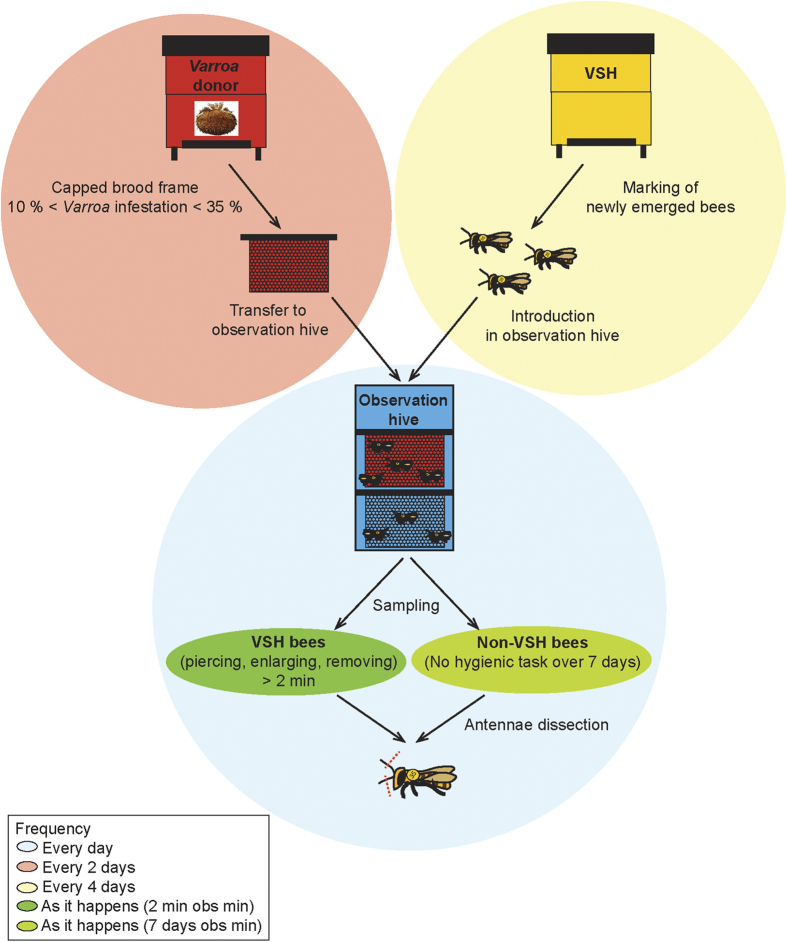
Schematic illustration of the behavioural assay and sampling of VSH and NVS bees. **** This assay was designed to sample adult honey bees performing, or not, VSH tasks. *Varroa*-infested frames (red) were placed in an observation colony (blue), together with marked newly emerged bees from a VSH colony (yellow). Everyday, honey bees that were seen performing VSH tasks (opening cells, enlarging openings, or removing brood from infested cells) for more than 2 minutes were sampled while they were still undertaking the behaviour. Honey bees that had not performed any VSH-related tasks over the course of 7 days of observation were considered to be non-VSH bees.

Using a bioassay performed in an observation hive, a total of 44 VSH bees of known age, and for which the VSH behaviour was described in detail, were collected (Fig. 1).

**Figure 2:**
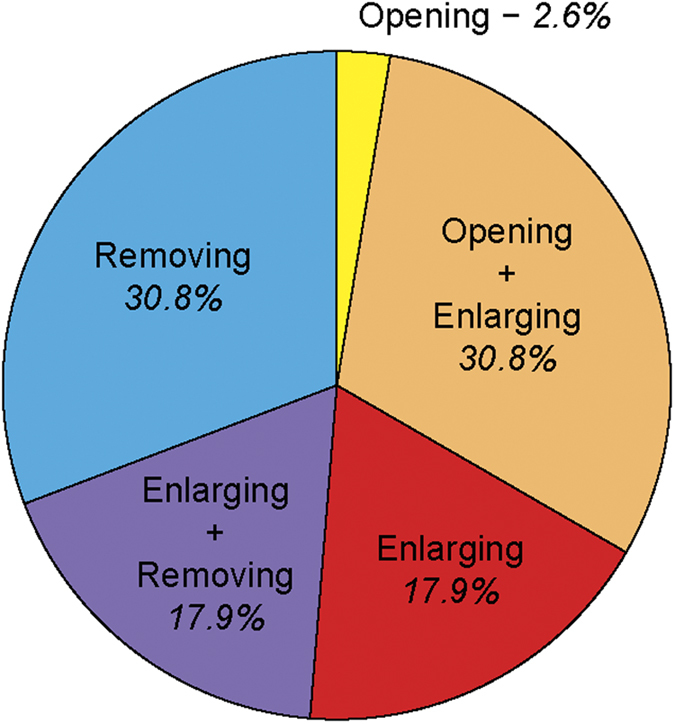
Frequency of the different components of VSH behaviours. **** Proportion of bees performing one or more of the three behaviours associated with VSH: opening capped cells, enlarging the openings, and removing brood from cells infested by *Varroa*.

The average age of VSH bees was 11.13 ± 3.7 days, with the youngest being 6 days old, and the oldest 18 days old. VSH bees were observed performing the 3 types of behaviours associated with VSH: opening or enlarging the opening of capped brood cells infested by *Varroa,* and removing brood from infested cells (Fig. 2). Approximately half of the sampled bees were seen performing more than one type of task (opening and enlarging, or enlarging and removing), the other half was mainly composed of enlargers and removers (Fig. 2). Very few bees were considered as solely openers, and no bees were seen performing all three behaviours. This most probably results from the design of the study, which involved sampling VSH-bees after they had been observed performing any VSH-related task for 2 minutes. This duration is longer that the time required to open a cell, but shorter than the time required to accomplish the full set of the three VSH behaviours.

### Virus detection in the antennae of VSH and non-VSH bees

Differences in the antennal transcriptomes of VSH and non-*Varroa* sensitive (NVS) bees were identified using RNA-seq analysis. Age-matched samples from VSH and NVS bees were analysed (see Supplementary Table S1 online). Four RNA libraries were generated for each behavioural group (VSH and NVS). The total number of unique aligned reads ranged from 13.8 to 43.8 million, of which around 74% were unambiguously and uniquely aligned to the *A.mellifera* genome assembly 4.5 (see Supplementary Table S2 online). The percentages of alignment were very similar between the two phenotypic groups suggesting an absence of bias in the sequencing data. Raw and processed genomic data are available from National Center for Biotechnology Information (NCBI) Gene Expression Omnibus (GEO) database with the accession number GSE63613.

**Figure 3:**
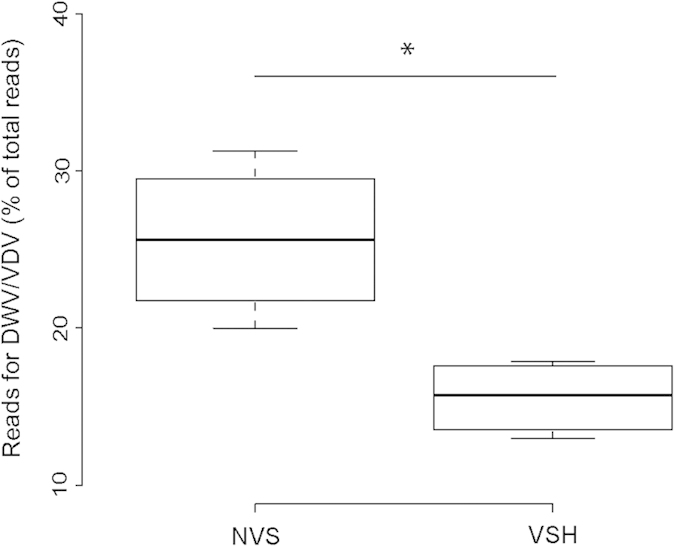
Relative abundance of Deformed wing virus and *Varroa destructor* virus (DWV/VDV) in the antennae of VSH and NVS bees. Virus abundance is expressed as a proportion of the total number of reads obtained by RNA-seq for each sample (n = 4 for each group). * denotes significant differences after Mann-Whitney tests (p = 0.014).

RNA-Seq analysis was first used to quantify contaminants amongst the honey bee transcripts, including viral RNAs. The 7 most-common honey bee viruses were screened (Sacbrood virus, SBV; *Varroa destructor* virus, VDV; Black queen cell virus, BQCV; Acute bee paralysis virus, ABPV; Deformed wing virus, DWV; Kashmir bee virus, KBV; Chronic bee paralysis virus, CBPV). Only DWV and VDV were detected in antennae, and represented 12.97 to 31.27% of the total reads. This proportion of reads attributed to viruses was used as an estimate of the sum of DWV and VDV abundances in the antennal samples. Viruses of the DWV/VDV complex were significantly more abundant in NVS antennae than in VSH antennae (Mann-Whitney test, p = 0.014 – Fig. 3).

From the nurse and forager bee samples (n = 3) included for comparison with VSH and NVS bee samples (see below), DWV and VDV were detected in one nurse and one forager sample only (7.5 and 28.8% of the total reads, respectively).

### Differential gene expression in the antennae of VSH and non-VSH (NVS) bees

A total of 258 coding transcripts were found to be differentially expressed (173 up- and 85 down-regulated) in the antennae of VSH as compared to non-VSH (NVS) bees at an adjusted P-value < 0.05 (see Supplementary Table S3 online). The magnitude of the log-fold change ranged from 0.43 to 3.53 for the under-expressed genes and from 0.42 to 8.14 for the over-expressed genes.

Genes that were found to be differentially expressed in the antennae of VSH and NVS bees were explored using ontological classification provided by the *Apis mellifera* reference sequence entry in the NCBI and the DAVID databases. Gene Ontology analysis revealed that the ‘defence response’ category (defined as reactions triggered in response to the presence of a foreign body or the occurrence of an injury, which result in restriction of damage to the organism attacked or prevention/recovery from the infection caused by the attack) is enriched in the VSH bee antennae (p = 0.014). This category contained 5 genes, 4 of which are under-expressed (*Def1*, *Def2*, *Mjrp1*, *GB51223*) and one is over-expressed (*18-w*). ‘Oxygene transporter activity’ was also slightly enriched (p = 0.042), with 3 over-expressed genes in VSH bees (*Hex110*, *Hex70a*, *PPO*).

Molecular processes and/or biological functions could be attributed to 180 genes and grouped in 9 main functions, according to the GO functions attributed to the *Drosophila* homolog (Table 1). Metabolism (general metabolism and oxidative phosphorylation) was the most strongly represented biological function (27.5% of the genes). ‘Redox metabolism’ was especially well-represented; 12 genes related to this metabolism function were over-expressed (*Pxd*, *Cyp4g11*, *CYP336A1*, *PPO*, *GMCOX6*, *GMCOX13*, *GMCOX5*, *GB48022*, *GB45188*, *GB51729*, *GB41912*, *LOC726337*), and 3 were under-expressed in VSH bees compared to NVS bees (*MsrA*, *GB52087*, *GB41212*). Other functions well represented included genes related to motor activity or to neuronal processes, with 11 genes associated with odour perception and olfactory information processing. Interestingly, these functions related to ‘metabolism’, ‘motor activity’ and ‘neuronal processes’ tended to be over-expressed in VSH bees as compared to NVS bees.

### Overlap between VSH/NVS and forager (FOR)/nurse (NUR) bees gene sets

To further characterise the antennal transcriptomic profile of VSH bees, the VSH bee gene expression profile was compared to the gene set associated with foraging versus nursing activities. Nurses and foragers from the different colonies were analysed providing 2 or 3 RNA libraries per phenotype (23 to 82.6 million unique mapped reads and nearly 74.0% of those unambiguously and uniquely aligned to the bee genome assembly 4.5). Again, those alignment percentages were highly similar between nurses and foragers and to the VSH and NVS groups (see Supplementary Table S2 online). A total of 426 coding transcripts were found to be differentially expressed between the antennae of forager (FOR) and nurse (NUR) bees (see supplementary Table S4 online). Gene Ontology analysis revealed that the ‘secondary metabolites biosynthesis, transport and catabolism’ category is enriched in forager bee antennae (p = 0.018). This category contained 5 cytochrome P450 genes, 3 of which were under-expressed (*Cyp301a1*, *Cyp49a1*, *Cyp4g11*) and 2 of which were over-expressed in foragers compared to nurses (*Cyp6bd1, Cyp9q3*). The FOR/NUR gene set has 11 representatives involved in ‘redox metabolism’ (*PPO, GMCOX4, GMCOX6, GMCOX14, GB726471, GB725930, GB410227, GB410021, GB724890, GB552158, GB552157*), all of which are expressed at lower levels in foragers than in nurses. Molecular processes and/or biological functions could be attributed to 257 genes and grouped in 10 main functions, according to the GO functions attributed to the *Drosophila* homolog (Table 2).

**Figure 4:**
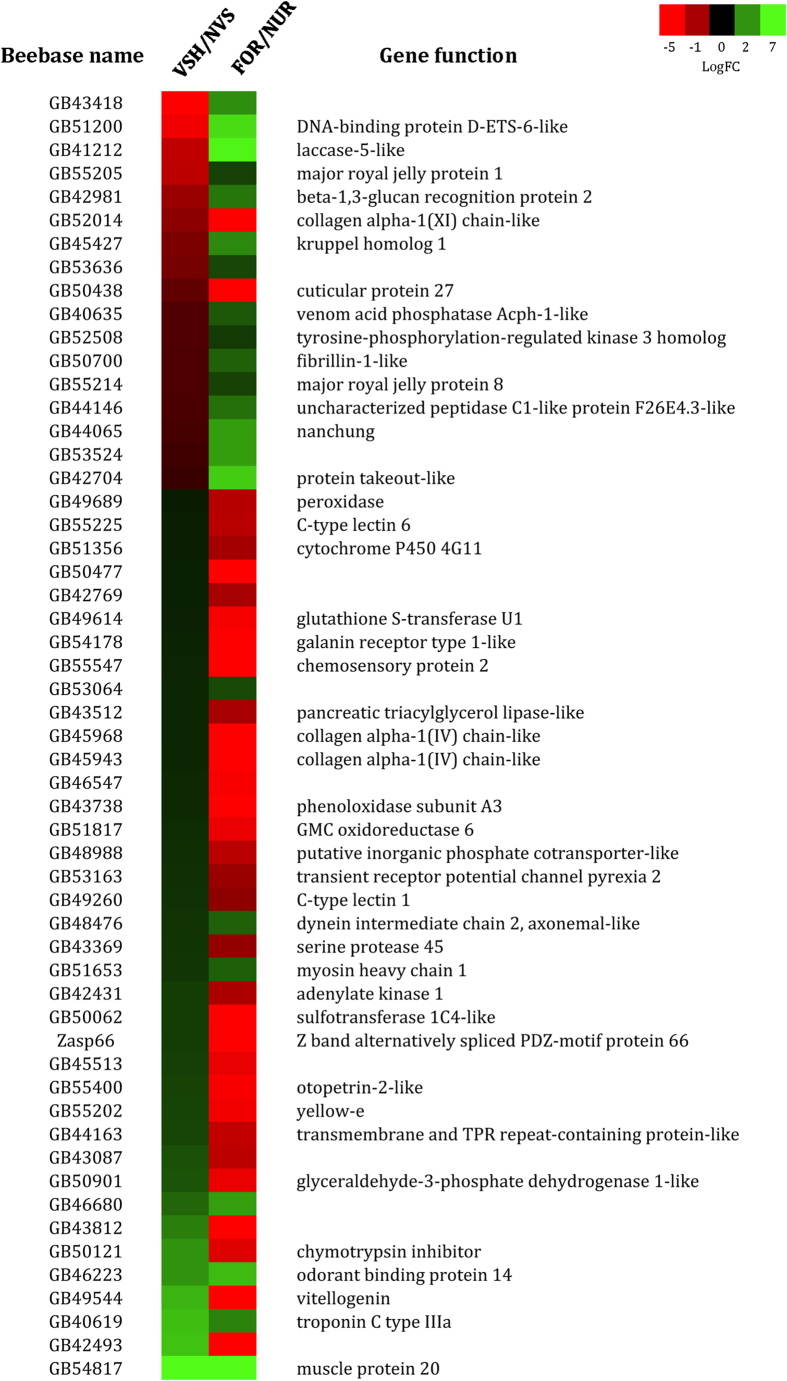
Heat-map of log fold-change (logFC) values for genes that are commonly regulated between VSH/NVS and FOR/NUR. **** Boxes in green indicate genes that are over-expressed in VSH or FOR, boxes in red indicate genes that are over-expressed in NVS or NUR.

57 genes overlapped between the VSH/NVS and FOR/NUR phenotypes. The list of these 57 genes is provided in Fig. 4. This overlap was 4 times higher than what would be expected by chance alone (hypergeometric test, p < 0.0001).

The directional biases of the overlapping genes between VSH/NVS and FOR/NUR phenotype were also highly significant (X^2^ = 27.52, p < 0.0001 - Table 3),with a larger proportion of genes over-expressed in VSH being also over-expressed in nurse antennae, and *vice versa*.

## Discussion

Comparisons of antennal transcriptomes using RNA-Seq revealed 258 genes that were differentially expressed between VSH and non-VSH bees. This suggests that the origin of VSH behaviour is at least partly related to shifts in antennal gene expression, strongly supporting the hypothesis that antennae play a critical role in this behaviour.

Amongst these genes were several genes related to chemical sensing, all of which were expressed at higher levels in VSH bees. This points to differences in the chemical sensing capabilities of VSH and non-VSH bees. Of particular interest is the olfactory binding protein gene *OBP3.* While this gene is not expressed only in antennae^29^, as a carrier of odorant molecules, it is likely to contribute to the olfactory sensitivity of VSH bees. This same gene was identified also as a gene of interest in a transcriptomic study investigating the central nervous system of honey bees and its contribution to the origin of VSH behaviour^20^. Interestingly, *OBP3* in the brain is regulated in the opposite direction to the antennae; while it is expressed at higher levels in the antennae of VSH bees than in non-VSH bees, the opposite is true in the brain. Taken together, these data highlight *OBP3* as a strong candidate for involvement in the molecular underpinnings of VSH behaviour. The clear differential expression of 11 olfactory-related genes (*Trh*, *OR85b-like*, *CSP2*, *NT-7*, *OBP3*, *OBP14*, *GB44647*, *Pyx2*, *GB41230*, *GB50377*, *GB43812*) suggests that VSH bees express a unique form of odour-guided behaviour. This result is in accordance with findings of several other studies that have investigated genes associated with hygienic behaviour, or with the trait of resistance to *Varroa* of honey bee colonies. Three studies have used transcriptome analyses^20^,^30^,^31^, one has focused on the proteome^32^, and two studies have examined genetic mapping using QTL analyses^19^,^33^. In total, 17 genes of interest in the present study overlap with genes of interest identified in earlier reports (Table 4). Of these genes, 5 have functions related to neuronal function or olfactory information processing (*GB52992*, *GB41900*, *GB1356*, *GB55511*, *GB53371*).

Rather unexpectedly, almost 30% of the genes expressed differentially in the antennae of VSH and non-VSH bees have functions related to metabolism. For example, several genes involved in ‘redox metabolism’ or ‘oxidative phosphorylation’ functions were up-regulated in VSH bees, suggesting a higher metabolism in the antennae of VSH bees in comparison to non-VSH bees. Interestingly, redox metabolism and oxidative phosphorylation were also strongly represented in the differential gene expression patterns of forager versus nurse bee antennae, with 11 genes showing higher levels of expression in nurses. Three genes are commonly up-regulated in VSH and nurse bees. For example, *GB51356*, which encodes a cytochrome P450 (4G11), was found by Le Conte *et al.* to be differentially expressed also in the brains of VSH and non-VSH bees^20^ (Table 4). The function of this cytochrome in bees is still uncertain, but in other insects it has been strongly implicated in metabolic pathways linked to olfaction^34^. One of the two GO enriched functions in VSH bees relates to oxygen transporter activity, with up-regulation of the representing genes. As cognitive and behavioural tasks in insects are known to increase both carbohydrate metabolism and oxygen consumption^35^,^36^, the over-representation of genes involved in metabolism may reflect metabolic loads associated with the detection and removal of *Varroa*-parasitised brood. The over-expression in VSH bees of 24 genes involved in motor activity may also be indicative of higher levels of antennal activity in VSH bees. However, these effects could also be related to differences in viral loads in the antennae of VSH and non-VSH bees.

Interestingly, two viral species (VDV and DWV) were identified in the antennae. The proportion of reads attributed to viral RNA relative to genomic RNA indicates that the abundance of VDV/DWV is lower in the antennae of VSH bees compared to non-VSH bees. Bees comprising these two groups originated from the same colony, and were raised together within the observation hive. They had all been subjected to same genetic, social and environmental conditions prior to sampling. The difference in virus abundance is particularly interesting with regards to the GO enrichment related to ‘defence response’ detected in the antennal DEG profile. Most genes attributed to this category were under-expressed in VSH bees (e.g. *Def1* and *Def2*). In mammals, defensins play an active role in antiviral defence and their expression can be induced by viruses in target cells that produce factors to prevent viral replication in the tissues^37^. Defensins are abundant in the haemolymph of insects^38^. Considering the high abundance of DWV in the antennae of non-VSH bees, it is likely that the over-expression of defence response genes in these bees is a sign of activation of antiviral response to DWV infection. DWV can be detected in the central nervous system^39^ and evidence suggests that DWV infection can interfere with the molecular mechanisms of learning^40^. Other viruses are also known to affect sucrose responsiveness in the honey bee^41^, and to change gene expression in the brain^42^. More generally, in vertebrates, viral infections can cause defects in the function of the nervous system, including a wide range of impairments in cognitive and motor function, but also of social behaviour^43^,^44^,^45^. Thus, it is possible that increased DWV infection in the antennae of NVS bees prevents these bees from developing VSH behaviour, perhaps by impairing some cognitive functions or altering the perception of olfactory cues. Interestingly, proteomic studies investigating the basis of worker reproduction also identified higher virus loads, primarily DWV, in the haemolymph^46^, ovaries and brains^47^ of sterile bees as compared to reproductive bees. This suggests that the extent of virus infections of individuals may ultimately influence behavioural performance: only healthy individuals with limited and/or controlled infections are able to achieve complex tasks within the colony, or to remain nurse bees (highly infected bees are known to accelerate their behavioural development toward foraging activity^48^,^49^,^50^).

This study also described a significant overlap between the VSH/NVS and nurse/forager gene sets, with VSH bees having a more similar profile to nurse bees than to forager bees. This was confirmed by the expression pattern of the transcription factor *Kr-h1*, which is up-regulated in foragers in comparison to nurses^51^,^52^(this study). This gene was expressed at lower levels in VSH as compared to non-VSH bees. These results confirm findings suggesting that hygienic behaviour is not related to foraging ontogeny^53^. They are consistent also with studies showing no overlap in the brain gene expression profile of VSH and forager bees^20^.

As VSH bees display a similar gene expression pattern to nurse bees this suggests that VSH bees probably spend more time in inside-hive activities. The age-range of VSH bees observed in this study confirms this hypothesis and would attribute VSH bees to the caste of nurse bees, as has been suggested for bees performing general hygienic behaviour^19^,^54^,^55^. It is possible that VSH bees are a sub-population of nurse bees that - like nurses - give care to the brood, but that – unlike nurses - could be specialised in caring for capped brood. It has been shown that high levels of brood pheromone are found in uncapped late-instar larvae^56^ and that such levels trigger capping of the brood cells^57^. Interestingly, this high level of brood pheromone seems to inhibit genes that are over-expressed in VSH bees^20^, suggesting that VSH bees are not involved in capping of late-instar larvae.

Despite this potential specialisation, nurse and VSH bees most probably share common requirements with respect to sensory detection capabilities adapted to the brood environment, including detection and processing of odours related to brood, wax and possibly mites, as well as the ability to monitor humidity and temperature variations^58^. Accordingly, amongst the commonly regulated genes between VSH and nurse bees, are 3 genes that participate in chemical sensing (*Nanchung*, *CSP2*, *OBP14*). Together with olfactory receptors (ORs), chemosensory proteins (CSPs) and olfactory binding proteins (OBPs) play a critical role also in the detection of chemical stimuli^59^.

Understanding complex social behaviours such as VSH behaviour requires investigation at multiple levels by combining methods of investigation. Transcriptomic studies offer the opportunity to capture genomic signatures of a particular behaviour, when experimental procedures allow for snap-shots of the behaviour to be taken through periods of thorough observation. Genetic mapping techniques, on the other hand, such as QTL analyses, help define genomic regions associated with variation in a phenotypic trait. Multi-level analyses are most likely to provide the key to identifying behaviourally relevant genes that are appropriate targets for the commercial development of honey bee lines that show long-term survival in the face of the parasite *Varroa*. Such analysis would also improve our knowledge on the evolution of this collective defences and its molecular basis.

## Material and Methods

### Behavioural assay and sample collection

The behavioural component of this study was performed using hybrid *Apis mellifera* (*ligustica* and *carnica*) honey bee colonies located at the Department of Zoology of the University of Otago (New Zealand), from February to April 2014.

A behavioural assay was designed to identify and to sample adult honey bees performing VSH tasks, as well as bees that do not perform such tasks. A three-frame observation hive was established, with a mated queen and approximately 1,500 adult bees randomly collected from one of the laboratory colonies. The observation colony was maintained in a dark enclosure and temperature was regulated (20–30 °C). Every 4 days, a frame containing late instar pupae was collected from a colony selected for its good VSH performances (BettaBees Ltd, New Zealand). In this donor colony, Varroa infestation level was kept low (<5 mites/100 bees phoretic infestation at the start of the experiment). Each sampled frame was maintained in an incubator (34 °C, uncontrolled humidity) and newly emerged adult bees were individually marked with an identity tag including a number (Ecroyd Ltd) and a paint mark (Chromacryl) on the thorax (Fig. 1). A total of 1,300 newly emerged bees were marked and introduced into the observation colony, in groups of 300-400 bees every four days. Tagging and paint marking enabled individual bees to be identified over several days of observation, and the age of all marked bees to be determined.

Observations began when the oldest marked bees were 7 days old. Every 2 days, a frame containing capped brood, 10 to 35% of which were infested by *Varroa* (Fig. 1), was placed in the observation hive. Everyday thereafter, from 9 am to 6 pm, two experimenters watched the behaviours of marked bees (one person on each side of the observation hive). “VSH bees” were designated as marked bees performing VSH-related tasks for more than 2 minutes - i.e. opening a capped cell and/or enlarging a pre-opened cell and/or removing the contents of a cell (Fig. 1). Only those bees that were the first to start removing a developing bee were included in the analysis; bees removing developing bees that had already started to be eaten were discarded in an effort to avoid any confusion between *Varroa*-parasitism and death as potential cues for removal. “Non-VSH bees” were designated as marked bees that were never seen to perform VSH related tasks (Fig. 1). These bees were included as negative controls. To ensure the absence of VSH-related tasks, control bees were observed for a minimum of 7 days before they were identified as non-VSH bees. For each sampled VSH bee, effort was made to sample one non-VSH bee, of similar age. A total of 81 marked adults of known age were collected using this procedure. Nurse and forager bees were collected from three ‘standard’ hives for comparison with VSH and non-VSH bees. Nurse bees were identified as adult bees seen with their heads in brood cells containing young larvae, while forager bees were sampled at the hive entrance and identified as adult bees returning to the colony with loads of pollen on their hind legs.

VSH, non-VSH, nurse and forager bees were collected from hives using soft forceps and immediately cold anesthetised. The flagellum from each antenna was quickly removed (Fig. 1) and the two antennal flagella from each bee were placed in a tube and flash-frozen in liquid nitrogen. All samples were stored at –80 °C until further processing.

### RNA isolation and transport

For transcriptomic analyses, total RNA extraction was realised on 4 pairs of antennae. To reduce a potential age effect on the transcriptome^60^, antennae were pooled only if the age of the bees from which they were isolated did not exceed 3 days of difference between the four bees. In total, 8 pools of antennae were constituted for VSH and non-VSH bees (see Supplementary Table S1 – RNA extraction pools); VSH and non-VSH groups were also matched by age.

For each sample of 4 pairs of antennae, total RNA was extracted and purified using a procedure modified from^28^. The 4 pairs of antennae were placed in a 5 mL plastic CryoS tube (Greier Bio One) and crushed for 30 s in 500 μL of trizol and 5 μL of carrier RNA (5 ng/μL – Invitrogen). Samples were incubated for 5 min at RT and after transfer into 1.5 mL tubes, 100 μL of chloroform was added. Samples were hand-shaken for 15 s and then left for 3 min at RT. They were then centrifuged at 12,000 rcf for 15 min at 4 °C. The upper phase was gently pipetted out. Total RNA was extracted following the Purelink Micro kit (Invitrogen) instructions, eluted twice in 12 and 10 μL nuclease-free water and stored at −80 °C until further processing. RNA yield and concentration were measured by Qubit (Life Technologies) and RNA integrity and quality were assessed using a BioAnalyzer (Agilent Technologies).

Transcriptomic analyses were performed in France. To prepare the samples for shipping, the total volume of each sample was added to an RNA Stable tube (Biomatrica). The mixture was dehydrated for 1 hour using a SpeedVac (Eppendorf Concentrator Plus). Dry samples were then shipped to France by post.

Upon reception in France, RNA integrity and concentration was estimated by a BioAnalyzer (Agilent Technologies). Transcriptomic analysis was performed on 4 replicates for each group (VSH, non-VSH), after combining two pools of 4 pairs of antennae to form one sample (8 pairs of antennae sampled on bees of the same age (±3 days)) (see Supplementary Table S1 – Transcriptomic samples). Similarly, 2 and 3 replicates of 8 pairs of antennae were analysed for the forager and nurse groups, respectively.

### RNA-seq analyses and sequence alignments

RNA sequencing and analysis of the RNA-seq data were carried out at the Institut de Genomique Fonctionnelle (Montpellier, France). The RNA-seq libraries were prepared using the TruSeq Stranded mRNA Sample Preparation kit according to the manufacturer’s instructions (Illumina). Qualitative and quantitative library validations were performed on a DNA 1000 Agilent chip as well as by quantitative PCR using SYBR Green (Applied Biosystems 7500).

Clusters were generated on a flow-cell within a cBot using the Cluster Generation Kit (Illumina) and libraries were sequenced on a HiSeq2000 using a SBS (Sequence By Synthesis) technique (Illumina). Image analysis was performed with the HiSeq Control software (HCS) and base calling using the RTA software. Quality control was operated through the FastQC software. Potential contaminants were investigated with the FastQ Screen software; several animal and bacterial genomes, as well as honey bee virus genomes were tested.

TopHat2 (TopHat2.0.12 with Bowtie 2.2.3) was used to align RNA-Seq reads to the *A*.*mellifera* genome assembly 4.5, and aligned reads were counted using the HTSeq-count software (version 6.1p1), with GeneID (NCBI) identifiers. Mapping was also performed on sequences of honey bee virus genomes (Sacbrood virus: NC 002066.1, Deformed wing virus: NC 004830.2, Black queen cell virus: NC 003784.1, Acute bee paralysis virus: NC 002548.1, Kashmir bee virus: NC 004807.1, Varroa destructor virus: NC 006494.1 and Chronic bee paralysis virus: NC 010711.1).

### Statistical analysis

All statistical analyses and figures were generated in the R environment (version 3.0.2), and an α level of 0.05 was used for all statistical tests. Pools of 8 pairs of antennae were considered as the individual in all tests. For descriptive statistics, the mean and standard deviation of the data set considered are indicated (and separated by the ± sign).

Genes that had less than 15 (NUR vs FOR) or 20 (VSH vs NVS) cumulated tag occurrence in all samples were discarded from the analysis and all remaining data were normalised (Relative Log Expression – RLE). The R package EdgeR (version 3.4.0) was used to determine differentially expressed genes (adjusted P ≤ 0.05) between VSH and non-VSH bees (n = 4), as well as between FOR and NUR bees (n = 2 and n = 3, respectively). EdgeR^61^,^62^ is an exact test based on a negative binomial distribution. Due to high performance in the ability to uncover true positives, this package is recommended for differential expression analysis of RNA-Seq data^63^. The P-value was adjusted for multiple testing using the Benjamini-Hochberg procedure.

Comparison of virus abundance between VSH and NVS antennae was performed using a non-parametric Mann-Whitney test due to the low number of replicates in each group (n = 4, two-tailed).

DAVID 6.7 tool was used to determine the enriched functional groups, based on GO terms, within the complete list of gene expressed in antennae^64^. Exact hypergeometric probability test was performed to test the statistical significance of the overlap between the VSH/NVS and NUR/FOR gene sets^65^. We also determined whether the gene expression profile of VSH bees was more similar to nurses or to foragers using Chi-square tests with Yates correction.

## Author Contributions

F.M., C.A., A.R.M. and Y.L.C. conceived the study. F.M., C.A., D.S. and M.R. conducted the experiments and analysed the data. F.M., C.A., A.R.M. and Y.L.C. wrote the manuscript. All authors revised the manuscript.

## Additional Information

**How to cite this article**: Mondet, F. *et al.* Antennae hold a key to *Varroa*-sensitive hygiene behaviour in honey bees. *Sci. Rep.* **5**, 10454; doi: 10.1038/srep10454 (2015).

## Supplementary Material

Supporting Information

Supporting Information

Supporting Information

## Figures and Tables

**Table 1:** Summary of the main functions related to genes differentially expressed between the antennae of VSH and NVS bees.

**General function**	**Number of genes**	**Proportion of the DEG**	**GO functions**
**Motor activity**	(−) : 7	18.6%	Motor activity, muscle microtubule activity, muscle assembly
	(+) : 24		
**Neuronal processes**	(−) : 5	18.0%	Sensory perception (of smell and taste), neurotransmission, neuronal development and maturation, synaptic function
	(+) : 25		
**General metabolism**	(−) : 8	17.3%	Redox metabolism, carbohydrate metabolism, fatty acid metabolism, lipid metabolism
	(+) : 21		
**Transcription regulation**	(−) : 11	10.7%	Transcription factors, nucleosome assembly
	(+) : 7		
**Oxidative phosphorylation**	(−) : 5	10.2%	Oxidative phosphorylation
	(+) : 12		
**Structural**	(−) : 3	10.2%	Chaperones, cell adhesion, structural proteins of membranes
	(+) : 14		
**Immunity**	(−) : 4	6.0%	Antibacterial activity, immunity
	(+) : 6		
**Stress response**	(−) : 7	5.4%	Stress response, apoptosis
	(+) : 2		
**Transport**	(−) : 1	2.4%	Ion transport, carbohydrate transport
	(+) : 3		

The number of under (−) and over-expressed (+) genes in VSH bees as well as the total proportion of genes attributed to each function is indicated.

**Table 2:** Summary of the main functions related to genes differentially expressed between the antennae of FOR and NUR bees.

**General function**	**Number of genes**	**Proportion of the DEG**	**GO functions**
**General metabolism**	(−) : 25	19.1%	Redox metabolism, carbohydrate metabolism, fatty acid metabolism, lipid metabolism
	(+) : 16		
**Neuronal processes**	(−) : 18	14.0%	Sensory perception (of smell and taste), neurotransmission, synaptic function, neuropeptide, synapse organisation
	(+) : 12		
**Structural**	(−) : 23	13.5%	Structural component of membranes, cell adhesion, protein folding, extracellular matrix organisation
	(+) : 6		
**Transport**	(−) : 13	12.6%	Ion, lipid, carbohydrate and protein transport
	(+) : 14		
**Transcription regulation**	(−) : 4	9.8%	Transcription regulation, nucleosome assembly
	(+) : 17		
**Oxidative phosphorylation**	(−) : 16	9.3%	Oxidative phosphorylation
	(+) : 4		
**Cuticle structure**	(−) : 1	8.4%	Structure of cuticle, control of pigmentation of cuticle
	(+) : 17		
**Motor activity**	(−) : 5	5.1%	Motor protein, muscle assembly
	(+) : 6		
**Development**	(−) : 9	4.2%	Developmental proteins
	(+) : 0		
**Immunity**	(−) : 4	3.3%	Immunity, defence response, phagocytosis
	(+) : 3		
**Stress response**	(−) : 2	1.0%	Stress response
	(+) : 0		

The number of under- (−) and over-expressed (+) genes in FOR bees as well as the total proportion of genes attributed to each function is indicated.

**Table 3:** Gene expression overlap between VSH/NVS and FOR/NUR phenotypes.

	**Up-regulated in FOR**	**Up-regulated in NUR**
**Up-regulated in VSH**	7	32
	(28%)	(40%)
**Up-regulated in NVS**	16	2
	(12%)	(19%)

**Table 4:** Overlap between the VSH and other gene sets identified in different studies investigating the genetic basis of *Varroa* resistance.

**Beebase name**	**Previous GB**	**Gene description**	**Putative function**	**logFC**	**Transcriptomic**			**QTL**	
					**Gempe 2012** 31 **(Hygienic) Adults**	**Navajas 2008** 30 (***Varroa*****-surviving) Pupae**	**Le Conte 2011** 20 **(VSH) Brains**	**Tsuruda 2012** 33 **(VSH) Adults**	**Oxley 2010** 19 **(Hygienic) Leg, head, thorax**
GB51223	GB17538	Hymenoptaecin	Antibacterial activity	−2,035	−				
GB50423	GB10708			−1,261			−		
GB55335	GB18990	IQ and ubiquitin-like domain-containing protein-like		−1,117				X	
GB47475	GB19995	Protein lethal(2) essential for life-like	Stress response	−1,075					
GB10397	GB10397	Protein lethal(2) essential for life-like	Stress response	−0,555					
GB52992	GB14742	Agrin-like	Synaptogenesis	0,425	+				
GB41900	GB16304	Mind-meld	Maintenance nervous system	0,469	+				
GB49640	GB15912			0,471	+				
GB51356	GB11973	Cytochrome P450 4G11	Oxydoreductase	0,528			−		
GB55511	GB11204	Activin like protein at 23B-like	Neuronal function	0,534					X
GB46128	GB19001			0,543	+				
GB49614	GB15512	Glutathione S-transferase U1	Stress response	0,556	−				
GB49078	GB11812	Nose resistant to fluoxetine protein 6-like	Lipid metabolism	0,619					
GB46774	GB18056	DnaJ protein homolog 1-like	Chaperone	0,672		−			
GB55483	GB11358	Ig-like and fibronectin type III domain containing 2	Motor activity	0,872	+				
GB50609	GB19503	Heat shock protein Hsp70Ab-like	Chaperone	1,007		−			
GB53371	GB30242	Odorant binding protein 3	Odorant receptor	3,436			−		
