# Supplementary material for: Antennae hold a key to Varroa-sensitive hygiene behaviour in honey bees
Source: Sci Rep. 2015 May 22;5:10454. doi: 10.1038/srep10454 (PMC4441115; doi:10.1038/srep10454)
Supplement: Supporting Information [file srep10454-s1.pdf]

Antennae hold a key to *Varroa*-sensitive hygiene behaviour in honey bees

Fanny Mondet, Cédric Alaux, Dany Severac, Marine Rohmer,
Alison R. Mercer and Yves Le Conte

Supplementary information

Supplementary Table S1. Age range of bees sampled and analysed by RNA-seq
The table indicates the sample numbers for RNA extraction (n = 4 pairs of antennae per sample), for RNA-seq (n = 8 pairs of antennae per sample) and the corresponding ages for each sample.

Behaviour	Transcriptomic sample	RNA Extraction pool	Age (days)
VSH	1	1	7.5 ± 1
		2	7 ± 1.2
	2	3	9.75 ± 0.5
		4	9.75 ± 0.5
	3	5	12.5 ± 0.6
	4	7	17 ± 0.8
		8	17 ± 0
Non-VSH	1	1	7.5 ± 1
		2	7.5 ± 0.6
	2	3	9.5 ± 1
		4	9.5 ± 1
	3	5	12.5 ± 0.6
		6	12.25 ± 1
	4	7	17.5 ± 0.6
		8	17.25 ± 0.5

Supplementary Table S2. Summary of the DEG sequencing results

Sample	Total tags	Total filtered tags (Illumina)	Total aligned tags	Unique aligned tags
NVS1	21,484,068	19,732,362	14,058,950	13,821,128
NVS2	55,722,219	51,128,490	32,018,709	31,439,147
NVS3	77,733,897	65,659,295	44,631,194	43,806,021
NVS4	49,184,602	42,055,054	25,105,271	24,657,636
VSH1	19,536,465	17,840,513	13,934,725	13,708,453
VSH2	45,777,936	41,590,440	32,917,445	32,381,647
VSH3	50,057,199	42,303,321	30,866,091	30,271,604
VSH4	60,209,077	51,028,789	38,568,733	37,946,463
FOR2	103,455,670	89,933,520	84,125,568	82,586,953
FOR3	52,308,319	48,391,543	44,372,926	43,564,874
NUR1	78,188,150	68,124,252	58,293,569	57,357,144
NUR2	54,007,997	46,585,308	43,532,558	42,726,909
NUR3	27,540,844	25,586,890	23,419,313	22,977,132

Supplementary Table S3. List of genes differentially expressed in antennae of VSH and NVS bees

Corresponding *Drosophila* orthologs and log2 (VSH/NVS ratio) expression values of the fold change and count-per-million are shown.

(See corresponding excel file)

Supplementary Table S4. List of genes differentially expressed in antennae of FOR and NUR bees

Corresponding *Drosophila* orthologs and log2 (FOR/NUR ratio) expression values of the fold change and count-per-million are shown.

(See corresponding excel file)
